# Reviewing the therapeutic management of leprosy in primary care: demand case series referred to a University Hospital in the Midwest region of Brazil^☆^^☆☆^

**DOI:** 10.1016/j.abd.2020.09.005

**Published:** 2021-03-06

**Authors:** Pétra Pereira de Sousa, Ana Lúcia Maroccolo de Sousa, Marília Dalva Turchi

**Affiliations:** Instituto de Patologia Tropical e Saúde Pública, Universidade Federal de Goiás, Goiânia, GO, Brazil

**Keywords:** Epidemiology, Health services, Leprosy, *Mycobacterium leprae*, Public health

## Abstract

**Background:**

Leprosy still represents a negleted public health problem in Brazil. Early and adequate treatment of leprosy, carried out in a primary health network is essential to reduce morbidity and sequelae.

**Objective:**

To analyze the therapeutic management of leprosy patients referred from primary healthy services to a specialized service.

**Methods:**

An analytical retrospective study using medical records and the Notifiable Diseases Information System. Patients diagnosed with leprosy, referred to a specialized outpatient clinic, between 2016 and 2017, in Goiás state, were included. The treatment carried out in the primary health services was compared to the Ministry of Health guidelines.

**Results:**

Two-hundred twenty-five leprosy patients were included, of whom 33.3% were referred by leprosy reactions, 27.1% by sequelae, and 10.2% by suspected recurrence or reinfection. Reviewing the therapeutic management, 123 (54.7%) were considered inadequate, 92 (40.9%) adequate, and 10 (4.4%) inconclusive. Of the 200 multibacillary patients, 39.5% had adequate management. In contrast, 12 (85.1%) out of 14 paucibacillary patients had adequate management (χ^2^ = 11.43 and p < 0.001). Regarding the leprosy reactions and sequelae management, 56.9% and 19.5% were considered inadequate, respectively. There was no difference between the percentage of adequate or inadequate management when considering the Goiás health macroregions (χ^2^ = 7.23; 4 degrees of freedom; p = 0.12).

**Study limitations:**

Use of recorded data, with incomplete medical records and lack of patient follow-up.

**Conclusions:**

The study demonstrated the equivocal multibacillaryleprosy management conducted in healthy primary care, with an emphasis on leprosy reactions and sequelae. Training and monitoring the medical staff in the primary healthy services could reduce the morbidity and sequelae of leprosy.

## Introduction

Leprosy, also known as Hansen's disease, is a chronic infectious-contagious disease known to humankind since ancient times. It is characterized by predominant involvement of skin and peripheral nerves, caused by the *Mycobacterium leprae* (*M. leprae*), bacillus discovered in 1873 by Amauer Hansen.^1^ It can also present ocular, articular, ganglion, visceral, and bone marrow involvement.^2^, ^3^ Leprosy still represents an important public health problem, especially due to the stigmatizing sequelae and deformities. It is considered a neglected disease, strongly associated with unfavorable socioeconomic conditions.^4^

In 1991, the World Health Organization (WHO) approved a resolution to “eliminate leprosy as a public health problem” by the year 2000, with the elimination being defined as the reduction of prevalence to less than 1 case per 10,000 inhabitants.^5^ Despite the significant reduction in the worldwide prevalence of leprosy, the decreased detection of new cases has been slow, especially in developing countries, indicating transmission persistence and clinical management failures.^6^ In 2012, the WHO established a goal of “stopping global leprosy transmission by 2020”. However, there are still many challenges to overcome before reaching these goals.^7^

In 2018, three countries reported more than 10,000 new cases of leprosy, including India (120,334), Brazil (28,660), and Indonesia (17,017).^8^ These three countries account for approximately 81% of the new cases detected worldwide. In Brazil, after 13 years of decreased occurrence of leprosy, the number of new cases increased again in 2017, especially in the Midwest region.^9^ This region also showed one of the highest national prevalence rates, reaching 4.44 cases of leprosy/10,000 inhabitants.^9^

With the administrative and organizational decentralization of the Brazilian Unified Health System (SUS, *Sistema Único de Saúde*), patients notified with leprosy must be forwarded to the primary basic health unit closest to their homes, so that they have easier access to health care and a reduced chance of irregular or discontinuation of their treatment.^10^ This decentralization should be accompanied by actions aimed to qualify primary care professionals through continuing education and specialization courses, with reference centers taking on the role of conducting cases with a difficult diagnosis, clinical complications, and rehabilitation.^11^

There are still many obstacles to the implementation of leprosy control actions, hindering the action decentralization process, such as, for instance, the scarcity of adequate assessment instruments (such as the performance of sensitivity and bacilloscopy tests, skin biopsies) and the difficulty in analyzing the health care service routines.^12^, ^13^ To date, we have not identified any studies conducted in Brazil that assessed the difficulties in the therapeutic management of leprosy in primary health care, in line with the guidelines of the Ministry of Health (MH). The present study aims to analyze the adequacy of the therapeutic management of leprosy cases sent to a reference service in the Midwest Region of Brazil.

## Methods

This is an analytical retrospective study whose recorded data compiled from patients` medical charts of the Leprosy Outpatient Clinic at the University Hospital from January 1, 2016 to December 30, 2017 were evaluated.

Patients seen at the first consultation for diagnostic investigation of leprosy or for the management of complications, sequelae, or leprosy reactions, who had a referral letter from a primary public health service, without restriction of age, sex, or municipality of origin were included. Patients with a history of previous leprosy treatment at the Dermatology Outpatient Clinic of University Hospital, patients whose medical records did not include the treatments received for leprosy, their sequelae and leprosy reactions, or those whose data could not be retrieved from the leprosy notification/investigation forms of the Notifiable Diseases Information System (SINAN) were excluded.

The data were obtained from the patients’ medical records, letters of referral to the outpatient clinic, and the leprosy notification/investigation forms of SINAN (ICD-10 A30), made available by the Health Secretariat of the state of Goiás. As for the data collection procedure, initially, the Medical Archive Service at University Hospital provided a list with the nominal identification and medical record number of all patients treated at the leprosy outpatient clinic during the study period. The main researcher (PPS) reviewed the medical records and referral letters, and identified the cases that fulfilled the inclusion criteria.

The data were compiled in the Microsoft Excel® spreadsheet. The analyzed variables were: identification, sex, age, city of origin, initial diagnosis, the reason for referral, treatments performed at the service of origin, degree of physical disability, management in the specialized clinic, and final diagnosis.

The municipalities of origin were grouped inside the five health macroregions of the state of Goiás, namely: Northeast, Midwest, Mid-North, Southeast, and Southwest.^14^

The patients who were treated according to the Ministry of Health protocols had their management considered as adequate, as follows:^15^

Paucibacillary cases - Multidrug Therapy (MDT) with rifampicin and dapsone for 6 months.

Multibacillary cases - MDT with rifampicin, dapsone, and clofazimine for 12 months.

Type 1 leprosy reaction - Corticosteroids (first-line drug) and preventive therapeutic regimens for strongyloidiasis, diabetes, hypertension, and osteoporosis; alternative drugs: azathioprine, cyclosporine, when corticosteroids are contraindicated or cases are unresponsive to them.

Type 2 leprosy reaction - Thalidomide at varying doses, depending on the reaction severity, which may be associated with corticosteroids in cases of necrotic erythema nodosum leprosum, nerve trunk involvement (neuritis), orchiepididymitis, iritis or iridocyclitis, reactional hands, and feet; women of childbearing age and/or pregnant women – corticosteroids and/or clofazimine (or pentoxifylline). In childbearing age women, thalidomide can be prescribed if provided using two contraceptive methods, with one being a barrier method.

Patients treated differently from what is recommended were classified as receiving inappropriate treatment and those whose data were not possible to be recovered were classified as inconclusive.

The Operational Classification was used for the classification of cases: Paucibacillary (PB) and Multibacillary (MB), taking into account the bacilloscopy.^16^

Regarding the criteria for the diagnosis of leprosy reaction, the ones recommended by the Ministry of Health were considered, in which type 1 reaction is characterized by an exacerbation of previous lesions or the appearance of new lesions, making them edematous; also, ulcerations may occur, while nerve trunks can increase in volume and become painful. In the type 2 reaction, the most common clinical manifestation is erythema nodosum leprosum, characterized by the onset of erythematous and painful nodules and nerve thickening. Other clinical presentations are necrotic erythema nodosum leprosum and erythema multiforme.^2^ A mixed reaction is said to occur when the patient has lesions that are characteristic of both type 1 and type 2 reaction conditions, with these lesions occurring simultaneously or not.^17^

As for recurrence, leprosy cases that were regularly treated with official standardized and correctly indicated regimens, who were discharged after cure, and who once again showed new clinical signs and symptoms of active infectious disease and who did not respond to the anti-reactional treatment were considered as cases of recurring disease.^16^ Drug resistance, on the other hand, occurs when the patient, even when submitted to regular treatment, does not show clinical and laboratory improvement, confirmed by PCR techniques, currently performed by sequencing analysis, as *M. leprae* is not cultivated in vitro.^18^

Statistical analysis was performed using the Statistical Package for Social Sciences software v.21 (SPSS Inc., Chicago, Illinois, USA). Continuous variables were described considering the minimum and maximum values and the mean and standard deviation. Categorical variables were shown as absolute and relative values (percentages). The chi-square test was performed to compare categorical variables. Variable data potentially associated with inadequate management were evaluated. The significance level was established at p < 0.05.

The research project was approved by the Research Ethics Committee of the University Hospital (CAAE: 06122919.4.0000.5078).

## Results

**Figure 1:**
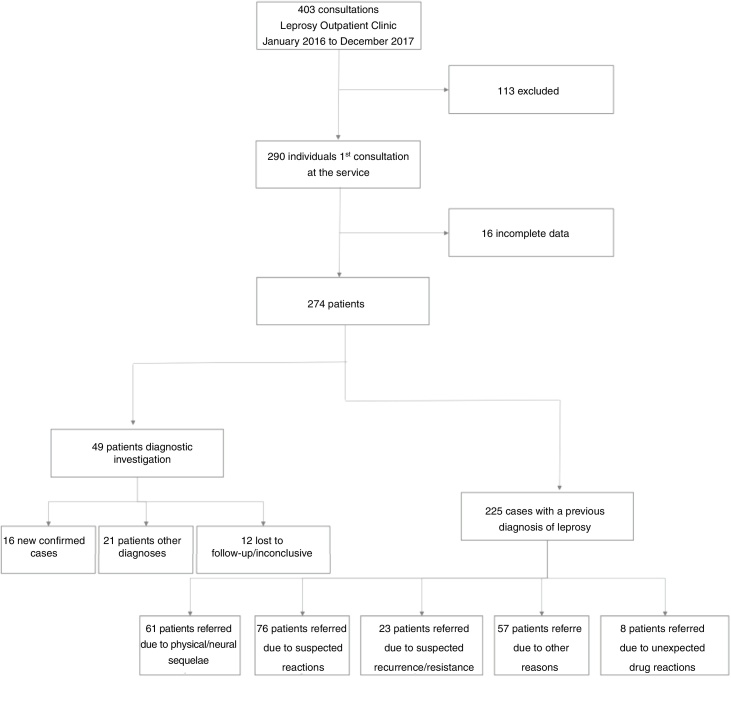
Flow chart of leprosy patients referred from primary healthy care to specialized service.

During the study period, 403 patients were treated at the Leprosy Outpatient Clinic, of whom 290 were attended by the first-time. However, 16 were excluded from the study because the evaluated data were not available. Fig. 1 shows the flowchart of patients selection and characterization, with regard to the reason for referral and the results of the diagnostic evaluation in the specialized outpatient clinic.

Of the 274 included patients, 163 were males (59.5%). The patients’ age ranged from 2 to 84 years, with a mean of 48.9 and a Standard Deviation (SD) of 15.9 years. Seven patients under the age of 15 years were seen, with three as suspected leprosy cases, one with type 2 leprosy reaction, one patient for post-treatment reassessment because a residual lesion persisted, another for a medical report, and an 11-year-old patient was referred due to suspected recurrence (inconclusive due to loss of follow-up). The three suspected cases were not ultimately confirmed as leprosy, receiving the diagnoses of atopic dermatitis and pityriasis alba.

Regarding the Health Macro-regions, 31 patients (11.3%) were referred from the Northeast region, 52 (19%) from the Mid-North region, 112 (40.9%) from the Midwest region, 56 (20.4 %) from the Mid-Southeast region, and 23 (8.4%) from the Southwest region.

The main reason for referring patients was type 2 leprosy reaction, in 52 (19%) patients, followed by suspected leprosy diagnosis in 49 (17.9%) patients. Of 49 patients who were referred as suspected cases, 16 had their diagnosis confirmed, with a prompt notification, and 12 were lost to follow-up. Twenty-one patients had other diagnoses: macular amyloidosis, Dupuytren's disease, pityriasis alba, systemic sclerosis, drug reaction, lichen simplex chronicus, pityriasis versicolor, pellagra, pre-neoplasms/neoplasms of the skin (Table 1).

**Table 1:** Final diagnosis of 49 cases referred as suspected leprosy to the Dermatology Outpatient Clinic of University Hospital from January 2016 to December 2017.

Final diagnosis	n (%)
Leprosy	16 (33%)
Loss of follow-up	12 (24.5%)
Polyneuropathy	6 (12.2%)
Pityriasis alba	5 (10.2%)
Pre-neoplasms/neoplasms of the skin	3 (6.1%)
Macular amyloidosis	1 (2%)
Systemic sclerosis	1 (2%)
Drug reaction	1 (2%)
Lichen simplex chronicus	1 (2%)
Pityriasis versicolor	1 (2%)
Pellagra	1 (2%)
Dupuytren's disease	1 (2%)
Total	49 (100%)

In summary 225 patients had a confirmed diagnosis of leprosy. Of these, 210 (93.3%) patients were classified as multibacillary, 14 (6.2%) as paucibacillary and one was not classified. Thirty-two patients had neural sequelae, such as loss of thermal, tactile, painful sensibility, in addition to permanent paresthesia (degree 1 of disability) and 29 physical sequelae (degree 2 of disability), such as plantar ulcers, claw hand deformity, foot drop, bone resorption, totaling 27.1%. Twenty (8.9%) patients were referred for different reasons, for instance, lack of thalidomide in the public network or for the treatment of several dermatoses such as tinea corporis, pellagra, stasis ulcer, pityriasis versicolor, and many of these patients had already been discharged after leprosy cure. Eight (3.5%) patients required an evaluation due to reaction to polychemotherapy drugs.

Twenty-two (9.8%) patients were referred due to type 1 leprosy reaction. Two patients had type 1 and type 2 reactions. Seven (2.6%) patients were referred due to a positive bacilloscopy test, even after the end of treatment.

For the classification of patients regarding the adequacy of treatment, those referred as a suspected case and, therefore, who had not undergone any treatment, were excluded, as well as those referred for biopsy whose diagnosis had not yet been attained. For treatment management reviewing, 225 patients had a confirmed leprosy diagnosis; 92 (40.9%) and 123 (54.7%) of whom had adequate and inadequate treatment, respectively, and 10 (4.4%) patients were classified as inconclusive.

Of the 200 MB patients 79 (39.5%) had adequate treatment management. In contrast, 12 (85.1%) out of 14 PB pattients had adequate management (χ^2^ = 11.43 and p < 0.001). One patient was not classified on the notification form.

Table 2 shows the distribution of the cases evaluated regarding the adequacy of treatment, stratified by health macroregions of the state of Goiás. The comparison between the percentage of cases classified as adequate *versus* inadequate management did not show a statistically significant difference amongst the macroregions (χ^2^ = 7.23; 4 degrees of freedom; p = 0.12). Inconclusive cases were excluded from this analysis.

**Table 2:** Therapeutic management classification of patients with leprosy, referred to a specialized Dermatology Outpatient Clinic, stratified by origin from the previous health service (Health Macroregions of the state of Goiás).

Macroregions	Therapeutic management			Total
	Adequate n (%)	Inadequate n (%)	Inconclusive n (%)	
Northeast	12 (42.9%)	15 (53.6%)	1 (3.6%)	28
Mid-North	24 (53.3%)	19 (42.2%)	2 (4.4%)	45
Midwest	27 (31.8%)	54 (63.5%)	4 (4.7%)	85
Southeast	21 (46.7%)	21 (46.7)	3 (6.7%)	45
Southwest	8 (36.4%)	14 (63.6%)	0	22
Total	92	123	10	225

When analyzing the treatment management for leprosy reactions, out of 22 patients having type 1 leprosy reaction, 86.3% were treated inadequately, while of those 52 patients having type 2 leprosy reaction, this percentage was 78.9% (Table 3) (χ^2^ = 0.81; p = 0.37). Inconclusive cases and cases with concomitant type 1 and 2 reactions were excluded from statistical analysis.

**Table 3:** Treatment reviewing classification according to the type of leprosy reaction.

Leprosy reactions	Clinical management			Total
	Adequate n (%)	Inadequate n (%)	Inconclusive n (%)	
Type 1	2 (9%)	19 (86.3%)	1 (4.7%)	22
Types 1 and 2	1 (50%)	1 (50%)	0	2
Type 2	9 (17.3%)	41 (78.9%)	2 (3.8%)	52
Total	12	61	3	76

When the inadequate management was analyzed, 59.3% of the patients had leprosy reactions, followed by sequelae (physical and neural), with 19.5% (Table 4).

**Table 4:** Causes of inadequate treatment management of leprosy patients attended in healthy primary care.

Grouping	Inadequate management, n (%)
Inadequate management of reaction	70 (56.9%)
Inadequate management of sequelae	24 (19.5%)
Diagnostic error	16 (13%)
Inadequate replacement regimen	9 (7.3%)
Standard regimen for an extended time	4 (3.3%)
Total	123 (100%)

**Figure 2:**
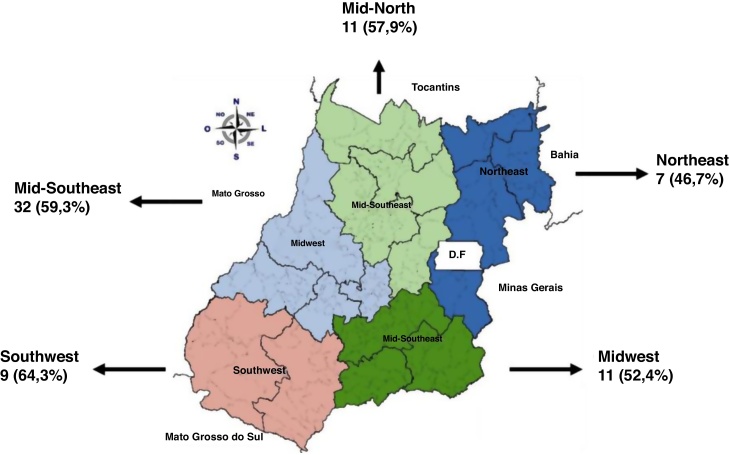
Inadequate management of reactions according to the Health Macro-regions of the State of Goiás, from January 2016 to December 2017.

According to the Health Macroregions of the state of Goiás, the percentages of inadequate management related to leprosy reactions were similar in all of them (Fig. 2).

Of the 206 patients whose degrees of disability were obtained from the SINAN, 116 (42.3%) had grade 0 disability, 55 (20.1%) had grade 1, and 26 (9.5%) grade 2. Nine (3.3%) patients were not assessed regarding the disability degree at the diagnosis. When comparing the degrees of disability attributted in the primary healthy care and in the specialized outpatient clinic, 13 patients who were initially classified as grade 0, were considered as grade 2. On the other hand, three patients who received a grade 2 classification in the primary care, were classified as grade 0 at the leprosy outpatient clinic of the University Hospital.

## Discussion

The present study allowed the identification of equivocal therapeutic management of leprosy patients referred from the primary health network, to a dermatology referral service in the state of Goiás, Brazil. In approximately 60% of the cases, there was some divergence of therapeutic management in disagreement with the Ministry of Health guidelines. Leprosy reactions and physical and neural sequelae were the main reasons for referral to a specialized outpatient clinic. A predominance of type 2 leprosy reactions was observed, with approximately 80% of cases having been conducted diversely from the Ministry of Health guidelines. This information draws attention to the risk of disease progression to physical disabilities (considered indicators of timely diagnosis, since patients with grades 1 and 2 constitute evidence of late diagnosis), social stigma, and worsening quality of life.^19^

Reactions type 1 and 2 of leprosy comprise very frequent complications, being responsible for the suffering and permanent disabilities of patients.^20^, ^21^, ^22^, ^23^ The diagnosis and management of leprosy reactions is a challenge for doctors, especially after polychemotherapy withdrawn. An early diagnosis and correct management are essential to prevent damage and reduce treatment abandonment.^20^ We emphasize that, in the present study, we observed a high percentage of inadequate management of leprosy reactions in all macroregions of the state. We also identified a high percentage of patients referred with physical and neural sequelae, from all the macroregions in the state. The presence of sequelae indicates a late diagnosis and/or inadequate management of the disease, signaling the need for specialized rehabilitation services in different regions of the state.^20^

The subdivision of the state of Goiás into health macroregions showed that the inadequacy of the performed treatments was similar in the different regions. It is noteworthy that the Midwest region encompasses cities such as Goiânia and its surroundings, that is, places where access to information and diagnostic methods would be more accessible. Therefore, the need for training programs aimed at professionals working in the primary health care network regarding the correct diagnosis and management of these patients is necessary.

Among the cases referred to the specialized service as suspected leprosy, approximately 30% were confirmed, suggesting that the primary care physician has difficulty in identifying these cases. Another fact that draws attention concerns diagnostic errors, in which cases notified as leprosy were excluded at the specialty clinic or even cases of leprosy reactions being treated as recurrence cases. It is known that in multibacillar patients, the bacilloscopy shows the presence of viable bacilli in the initial stages of treatment, while the proportion of granular bacilli increases with the continuity of treatment.^24^ Additionally, bacilloscopy does not become negative immediately, but it takes about a year to decrease by half a point in the bacilloscopy index (BI).^24^ That is, a patient with a high BI (especially those with an initial BI greater than 4+) will not show a negative BI at the time of discharge from the MDT.^25^ Despite the small percentage, seven patients were referred due to positive bacilloscopy after treatment, although the negative result is not a discharge criterion and takes a long time to occur.^24^

One patient was referred due to pregnancy while receiving MDT for MB leprosy. Treatment in these cases consists of the standard protocol with the use of multibacillary polychemotherapy and, if applicable, systemic corticosteroids to control the reactions. Although there is a recommendation to restrict the intake of these drugs in the first trimester of pregnancy, the benefits of treatment outweigh the risk. Thalidomide is contraindicated, as it is a teratogenic drug, and can be used by women of childbearing age only if the woman is using two contraceptive methods, with one being a barrier type.^26^

Examples of inadequate management, according to the Ministry of Health guidelines, constitute, for example, patients using prednisone for a very long time, without weaning, and without prophylaxis for strongyloidiasis or osteopenia/osteoporosis. Some of these patients had cushingoid facies, drug-related acne, dyslipidemia, hypertension, diabetes mellitus, striae, and cataracts.^15^

Leprosy learning during undergraduate medical school is mostly theoretical, with few cases seen by the students in medical practice. Therefore, primary care professionals often feel insecure in the management of leprosy patients, which can lead to diagnostic delays and iatrogenesis.^27^, ^28^

The present study has limitations, including those inherent to retrospective studies using recorded data. Among these, we can mention the incompleted data in the referral forms and medical records and the lack of patient follow-up, difficulting the clinical outcome assessment. Also, the study did not consider the year of diagnosis and the length of treatment prior to referral to the specialized service as variables that could be potentially associated with inadequate management.

Despite the limitations, the study showed a high percentage of leprosy patients referred to a specialized outpatient clinic whom had been managed in disagreement with the guidelines of the Ministry of Health, in all health macroregions of the state.^16^ We also emphasize the scarcity of studies evaluating the adequacy of the therapeutic management of leprosy by doctors in primary care, in other regions of the country, which can underestimate the magnitude of this problem.

Overall, in-person consultation at the tertiary level adds obstacles to the access, such as difficulties for intra and inter-municipal displacements, costs and delays in scheduling medical appointments. The training and supervision of primary care teams are essential to reduce problems related to the management of leprosy patients. In this context, the application of health information technologies (telehealth) makes it possible to expand the training of professionals and increase the resolution of leprosy treatment in primary care.

Different remote assistance and monitoring strategies could contribute to having leprosy patients evaluated by dermatologists, in referral centers, when necessary, using a more agile and interactive approach, with a consequent reduction in morbidity and sequelae.^29^, ^30^

## Conclusion

Leprosy reactions and physical/neural sequelae represented about 60% of the cases referred from the health primary care network to a tertiary referral center for leprosy, in Goiás. In a significant number of cases, the adoption of therapeutic management approaches was in disagreement with the guidelines recommended by the Ministry of Health. The study indicates the need to reinforce health education strategies for primary care professionals, as well as streamline the assessment of patients in tertiary centers, not necessarily through in-person appointments. The primary purpose of comprehensive care for the patient with leprosy is not only the reduction of the disease burden and its sequelae but also the correct diagnosis, to avoid unnecessary treatments and disease progression, which lead to physical disabilities.

The authors also suggest that prospective studies should be carried out to better assess the therapeutic management of leprosy patients treated at the primary basic care network, as well as the creation of specialized rehabilitation services in different regions of the country, aiming to prevent morbidity and sequelae.

## Financial support

None declared.

## Authors’ contributions

Pétra Pereira de Sousa: Conception and planning of the study; data collection and analysis; literature review; writing of the manuscript.

Ana Lúcia Maroccolo de Sousa: Conception and planning of the study; participation in the drafting of the manuscript; approval of the final version.

Marília Dalva Turchi: Planning of the study; statistical analysis and data interpretation; participation in the drafting of the manuscript; critical review of the manuscript.

## Conflicts of interest

None declared.
